# Evaluation of BCL2 and TNFα as mRNA biomarkers for monitoring the immune response in critically ill children

**DOI:** 10.1016/j.amsu.2018.10.024

**Published:** 2018-10-30

**Authors:** Ahmed Nabih El Shazly, Doaa Refaey Soliman, Shuzan Ali Mohammed, Rasha Mohammed Zakaria, Fatma Elzahraa Mohammed Awais

**Affiliations:** a Department of Pediatrics, Faculty of Medicine, Benha University, Benha, Qualubia, Egypt; b Department of Medical Biochemistry and Molecular Biology, Faculty of Medicine, Benha University, Benha, Qualubia, Egypt

**Keywords:** **HAI**, Hospital acquired infection, **MODS**, Multiple organ dysfunctions, **CARS**, Compensatory anti-inflammatory syndrome, **CDC**, Centers for disease control, **PELOD**, Pediatric logistic organ dysfunction, **OFI**, Organ failure index, **PICU**, pediatric intensive care unit, **TNFα**, Tumor necrosis factor alpha, **BCL2**, B-cell lymphoma 2, **qRT-PCR**, quantitative real time PCR, **cDNA**, Complementary DNA, **AUC**, Area under the curve, **ROC**, Receiver operating characteristics, TNFα, BCL2, Critical illness, Prediction of HAI, MODS, Quantitative real time PCR

## Abstract

**Background:**

Hospital acquired infection (HAI) and multiple organ dysfunctions (MODS) remain a leading cause of death in pediatric intensive care unit (PICU) despite the great efforts to control it.

**Objective:**

Our objective was to assess the mRNA of TNFα and BCL2 for prediction of HAI and/or MODS in our community.

**Patients and methods:**

Fifty children, admitted to PICU, were included in the study after exclusion of cases of end-stage renal failure, end-stage liver failure and congenital immune deficiency. Serial Blood samples were collected for complete blood count (CBC) and other routine investigations. Gene expression of (TNFα and BCL2) was quantified using quantitative real time PCR (qRT-PCR). Centers of disease control (CDC) criteria were used to detect HAI, and organ failure index (OFI). Pediatric logistic organ dysfunction (PELOD) and pediatric risk of mortality (PRISM) scores were used for follow up. The results were compared between the group who acquired HAI and who didn't. Gene expression was tested with a ROC curve to detect its ability to predict HAI.

**Main results:**

The overall complication (HAI and/or MODS) rate was 52%, Complicated cases had a significantly longer duration of stay in PICU (0.002) and in overall hospital stay (p = 0.013) and a higher death rate (p = 0.000). On day1; TNFα, BCL2 and lymphocytic count were lower in patients who developed complications (p = 0.02, p = 0.000 and p = 0.04, respectively), all had the ability to predict the complications with AUC (0.7, 0.8 and 0.67 respectively). On day 4: TNFα and BCL2 returned to normal levels while the lymphocytic count still lower in complicated cases, p = 0.001 and AUC = 0.73.

**Conclusions:**

TNFα and BCL2 on admission can predict HAI and MODS (AUC = 0.7 and AUC = 0.8), but were of no use in the follow-up, however, the lymphocytic count is a rapid, easy and cheap test to assess the immune state with a good predictive and follow up values.

## Highlights

• To our knowledge, our work is the 1^st^ to show mRNA biomarkers in acquired immunosuppressed critically ill Egyptian children.
• The current study provides a new evidence that immune suppression can be measured and opens the way for further studies.
• We mainly found that TNFα and BCL2 can predict HAI and MODS on admission but they were of no use in the follow-up.
• Lymphocytic count, a rapid, easy and cheap test, can assess the immune state with a good predictive and follow up values.

## 1 Background

Nosocomial sepsis and multiple organs dysfunctions (MODS) remain a major cause of morbidity and mortality in the pediatric intensive care unit (PICU) [1]. Early detection of patients at risk of sepsis or MODS would be of great help to provide them with suitable immunostimulatory drugs [2].

The immune system is massively activated with any critical insult, which is called severe inflammatory response syndrome (SIRS) and can lead to early mortality, then the patient tries to restore the immune balance by the compensatory anti-inflammatory syndrome (CARS), however uncontrolled CARS lets the patient be susceptible to infection and can lead to MODS and late mortality [3]. The CARS response includes: cutaneous anergy, endotoxin tolerance, lymphocytic apoptosis, decrease human leucocytic antigens (HLA) and anti-inflammatory mediator [4,5].

Critically ill neonates, children, and adults who die from nosocomial sepsis and MODS had prolonged lymphopenia (absolute lymphocyte count ‹ 1000 cells for more than 7 days) [6].

Apoptosis is not only a marker, but it has been shown to have a direct role in immune dysfunction, and that was obvious in murine studies, where prevention of apoptosis improved survival in sepsis [7]. Quantitative real-time PCR (qRT-PCR) is a new widely used technique that measures mRNA expression [8] and may detect enhanced lymphocyte apoptosis by measuring the mRNA of apoptosis-related biomarkers to identify patients at higher risk of nosocomial sepsis and MODS. B-cell lymphoma 2 (BCL2) gene, an anti-apoptotic gene, is one of the BCL2 group which regulates the intrinsic pathway of apoptosis [9]. It was used to detect the enhanced lymphocyte apoptosis and identify patients at higher risk of hospital acquired infection in previous researches [[10], [11], [12]].

Tumor necrosis factor alpha (TNFα), a proinflammatory cytokine, is a potent activator of many cell types such as macrophages/monocytes and NK cells and can induce cell survival or cell death by activation of the extrinsic pathway of apoptosis [13]. TNFα has been associated with mortality and hospital acquired infection (HAI) in several inflammatory situations. In a matched case-control study in Hinrichs, a combination of three biomarkers– CD3D, IL1B and TNF was the best predictor of post-operative sepsis in an adult cohort of patients, with a specificity of 90% and a sensitivity of 85% [14].

## 2 Aim of work

This study aimed to assess the ability of mRNA biomarkers (BCL2 and TNFα) to predict patients at risk of HAI, sepsis and MODS, in our community.

## 3 Patient and method

This is a cohort study, carried out in the Pediatric Intensive Care Unit (PICU) in Benha University Hospital in cooperation with the Medical Biochemistry and Molecular Biology Department, Faculty of Medicine, Benha University, during the period from April 2017 to October 2017.

**Inclusion criteria:** all patients admitted to PICU between 2 and 12 years old.

**Exclusion criteria:**

• End-stage renal disease requiring chronic dialysis therapy.
• End-stage liver disease: cirrhosis with evidence of portal hypertension,
• Congenital immunodeficiency.
• Readmitted cases.
• Referred patients.
• Patients stayed less than 48 h in PICU and 4 days in hospital (discharged, died or referred).

HAI was defined according to CDC criteria [15], as a localized or systemic condition resulting from an adverse reaction to the presence of an infectious agent or its toxin that was not present on admission to the hospital. An infection was considered an HAI if all elements of a CDC criterion were not present during the period of admission but were all present on or after the 3^rd^ day of hospital admission.

Pediatric logistic organ dysfunction (PELOD), organ failure index (OFI) scores were used to estimate the severity of cases of MODS in PICUs and to describe correctly the clinical course of illnesses observed in critically ill children [16]. Routine investigations were conducted to all cases on admission.

Approval of the study was obtained from the Ethical Committee of Scientific Research, Faculty of Medicine, Benha University. Written informed consent was taken from the parents of each child.

The work has been reported in line with the STROCSS criteria [17].

### 3.1 Sampling

Two venous blood samples (1 mL each) were taken from each subject at the first and the fourth days of their hospital admission. Venous blood samples were taken on (EDTA), mixed well, stored at −80 °C for further assessment of TNFα and BCL2 gene expression as follow:

### 3.2 Quantitation of gene expression by real-time PCR

Gene expression was assessed by **quantitative Real-Time PCR assay** (qRT-PCR):

1. **Total RNA Extraction:** Total RNA Extraction was performed using 100 μl EDTA whole blood specimen of each subject via purelink RNA Mini kit (Life technologies) according to the manufacturer instructions.
2. **Ultraviolet Spectrophotometric Quantification of RNA by nanodrop 2000 Spectrophotometer *(Thermo Fisher Scientific, Wilmington, USA).*** To ensure significance, A260 readings should be > 0.15. An absorbance of 1 unit at 260 nm corresponds to 40 μg RNA/ml. The ratio between the absorbance values at 260 and 280 nm gives an estimate of RNA purity; pure RNA has an absorbance ratio (260/280) of 1.9–2.3 [18].
3. Relative quantitation of mRNA of the respective genes by real-time PCR using syber green reagents on 2 steps:

#### 3.2.1 The first step qRT-PCR

The first step qRT-PCR was for conversion of RNA into complementary DNA (cDNA) in a Veriti™ Thermal Cycler (Applied Biosystems), using High-Capacity cDNA Reverse Transcription kit (Applied Biosystem, Foster City, USA). The RT master mix for reverse transcription of each subject contained 2 μl RT buffer (10X), 0.8 μl dNTPs mix (25X), 2 μl RT random primers (10X), 1 μl MultiScribe™ Reverse Transcriptase, 1 μl RNase inhibitor, 4.2 μl nuclease-free water. Then the PCR mix for reverse transcription of RNA into cDNA included 10 μl RTmaster mix (2X) and 10 μl Extracted RNA. The Thermal cycling conditions for RNA reverse transcription were primer annealing at 25 °C for 10 min, reverse transcription for at 42 °C for 15 min and inactivation at 85 °C for 5 min.

#### 3.2.2 The second step qRT-PCR

The second step qRT-PCR was for quantitation of CD203c and ST2L gene expression in a Stepone real time PCR system (Applied Biosystem, Singapore). Singleplex reactions were done. Non-templete controls were included in each run. This step was performed using SensiFAST™ Sybr Hi-Rox Kit (Bioline Reagents Ltd, United Kingdom). Human β-actin was the endogenous control housekeeping gene. Melting curve analysis was done in each run to confirm the specificity of real-time PCR assay. The primers used were as follow: BCL2 (271bp); FP: 5′-GCCAGCTGCACCTGACGCCCTTC-3′, RP:5′-CCGCATGCTGGGGCCGTACAGTT-3' [10], TNFα (138bp); FP: 5′-CTCCTACCCGAACAAGGTCA-3′, RP: 5′-CGGTCACCCTTCTCCAACT-3' [19] and β-actin (160bp); FP: 5′-GAATCCACTGGCGTCTTCAC-3′, RP: 5′-CGTTGCTGACAATCTTGAGAGA-3' [19].

The Singleplex PCR reaction mix for quantitation of gene expression contained 10 μl Maxima SYBR Green qPCR (2X, no ROX), 0.05 μl ROX solution, 1  μl FP, 1  μl RP, 2  μl cDNA and up to 20  μl nuclease-free water. The Real time thermal cycler conditions were as follow; initial denaturation for 10  m at 95 °C, 45 cycle; denaturation at 95 °C for 15s, annealing at 52 °C for 30s and extension at 72 °C for 30s.

### 3.3 Data analysis

**Fig. 1:**
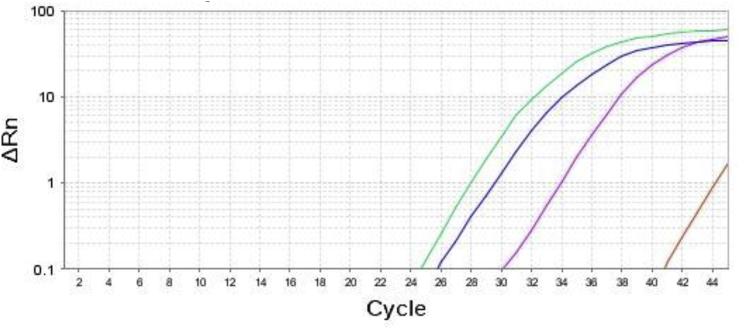
Amplification plot of the target genes (BCL2; blue curve, TNFα; green curve, β-actin; purple curve and non-template control; brown line). (For interpretation of the references to colour in this figure legend, the reader is referred to the Web version of this article.)

**Fig. 2:**
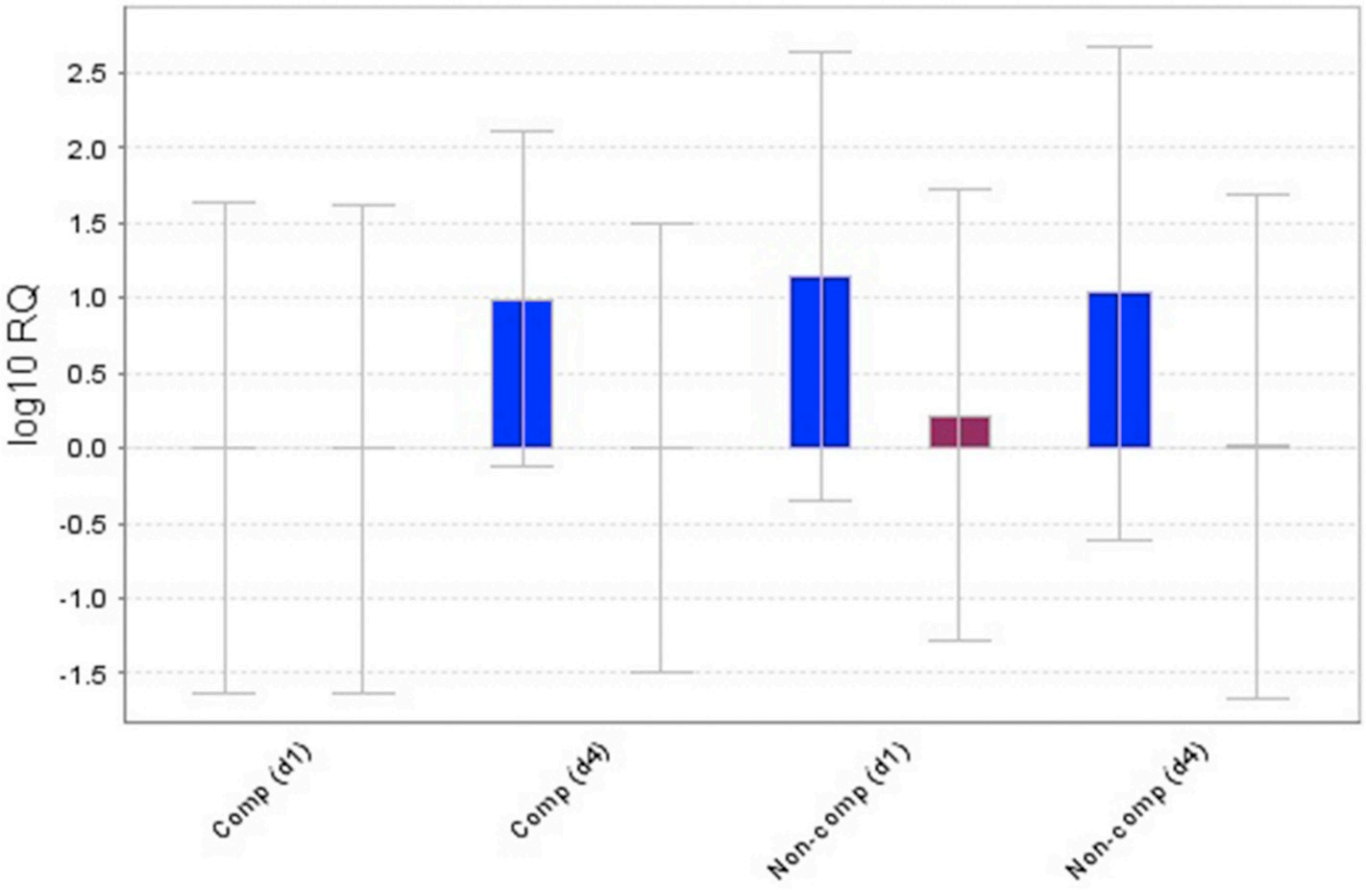
Gene expression plot of target genes normalized to β-actin gene as an endogenous reference gene (Blue bars indicate BCL2, Brown bars indicate TNFα however, β-actin had no bars in the graph). Comp: complicated, d: day. (For interpretation of the references to colour in this figure legend, the reader is referred to the Web version of this article.)

According to the RQ manager program, the data were produced as sigmoid shaped amplification plots in which the cycle number was plotted against fluorescence (when using linear scale). The samples of the control group were used as calibrators so the expression levels were set to 1. The relative quantities of human BCL2 and TNFα genes were normalized against the relative quantities of the endogenous control (human β-actin) so gene fold expression changes were calculated using the equation 2^−ΔΔCT^ [20]. As shown in Fig. 1, Fig. 2.

### 3.4 Statistics

The data were coded, entered and processed on the computer using SPSS (version 24). The results were represented in tabular and diagrammatic forms then interpreted. Cases were grouped and compared as follows: HAI vs. no HAI. Mean, standard deviation, frequency, and percentage were used as descriptive statistics.

Comparisons between groups were made using student-t test for continuous variables and the Chi-Square test (Χ^2^) for categorical data. Values of p < 0.05 were considered statistically significant.

Receiver operating characteristics (ROC) curve and areas under the curve (AUC) were calculated for each marker, as well as p-values that test the null hypothesis that the area under the curve equals 0.50. Correlations were performed using Pearson coefficient.

## 4 Results

This cohort study was conducted on 50 children admitted to PICU, who survived the early death (72 hr), samples were collected in the 1^st^ and 4^th^ days of admission, patients were divided into two groups according to their outcome; the first group (complicated), included children who acquired HAI and/or MODS and second group (Non-complicated) includes children who passed the critical insult without any complications.

Clinical data of those patients are described in Table 1 and Characters of complicated cases are described in Table 2.

**Table 1:** Clinical characteristics of the 50 patients regarding occurrence of complications.

Variable		Complicated (n. = 26)	Non-complicated (n. = 24)	p
		n. (%) or mean ± SD		
***Socio-demographic***				
*Sex: Male*		12 (46.2%)	14 (58.3%)	0.39
*Age (years)*		5.46 ± 2.72	3.75 ± 1.98	0.01^∗^
*Etiological admission cause*	Sepsis^*#*^	12 (46.2%)	12 (50%)	**0.02**^∗^
	Medical	4 (15.4%)	10 (41.7%)	
	Surgery & trauma	10 (38.5%)	2 (8.3%)	
***Past History***				
*Previous PICU admission*		14 (53.8%)	2 (8.3%)	0.001^∗∗^
*Comorbidities*	Cromosomal anomaly^†^	8 (30.8%)	2 (8.3%)	0.000^∗∗^
	Medical^‡^	8 (30.8%)	0 (0%)	
***Development-delay***		12 (46.2%)	4 (16.7%)	0.03^∗^
***PELOD***		7.54 ± 8.33	2.92 ± 4.78	0.02^∗^
***OFI***		1.92 ± 0.744	0.58 ± 0.776	0.000^∗∗^
***Treatments & invasive devices***				
*Ventilator*		22 (84.6%)	0 (0%)	0.000^∗∗^
*Blood transfusion*		18 (69.2%)	4 (16.7%)	0.000^∗∗^
*Central line*		10 (36.5%)	6 (25%)	0.30
*Urinary catheter*		8 (30.8%)	2 (8.3%)	0.048^∗^
*Corticosteroids*		6 (23.1%)	8 (33.3%)	0.42
*Antibiotics*		26 (100%)	14 (100%)	1
***Biological data 1***^st^***day***				
*Hemoglobin (g/dl)*		10.7 ± 1.6	10 ± 1.5	0.128
*Platelets (10*^*9*^*/L)*		262 ± 130	206 ± 137	0.145
*WBCs (10*^*9*^*/L)*		15 ± 10.7	14.7 ± 9	0.89
*Lymphocytes (10*^*9*^*/L)*		2.1 ± 1.4	3 ± 1.5	0.042^∗^
*Neutrophils (10*^*9*^*/L)*		12.2 ± 10.1	10.9 ± 7.8	0.611
*C reactive protein (mg/L)*		60.7 ± 67	31.16 + 40.09	0.067
***Biological data 4***^th^***day***				
*Hemoglobin (g/dl)*		11.3 ± 2	10.2 ± 1.7	0.03^∗^
*Platelets (10*^*9*^*/L)*		158 ± 101	246 ± 102	0.004^∗∗^
*WBCs (10*^*9*^*/L)*		13.1 ± 6.7	10.3 ± 4.5	0.091
*Lymphocytes (10*^*9*^*/L)*		2.1 ± 2	3.7 ± 2.4	0.012^∗^
*Neutrophils (10*^*9*^*/L)*		10.3 ± 6.7	6 ± 4	0.009^∗∗^
*C reactive protein (mg/L)*		74.5 ± 52.6	19 ± 21.7	0.000^∗∗^
***Outcome***				
*Stay in PICU (days)*		23.08 ± 26.97	4.92 ± 2.74	0.002^∗∗^
*Stay in hospital (days)*		31.54 ± 32.27	14 ± 7.66	0.013^∗^
*Death*		20 (76.9%)	0 (0%)	0.000^∗∗^

PICU: pediatric intensive care unit, ^#^: any infectious disease, ^†^: Cromosomal anomaly as Down syndrome, neuro-metabolic disorder), ^‡^: Medical as heart failure, bronchial asthma, epilepsy, otitis media, post-Corrosive, PELOD: pediatric logistic organ dysfunction, OFI: organ failure index, WBCs: white blood cells.

**Table 2:** Characteristics of complications.

Number of complicated patients 26 (52%) n.(%) or median (range)	
**Patients had HAI**	14 (28%)
**Delay of HAI (days)**	7 (4–10)
**Number of HAI attacks**	
1 HAI attack	6 (42.9%)
≥ 2 HAI attacks	8 (57.1%)
**Type of 1**^st^**HAI attack**	
Pneumonia	8 (57.1%)
Meningitis	2 (14.3%)
Device or wound associated	4 (28.6%)
**Causative organism**	
Gram –ve bacilli	5 (36%)
Klebsiella	3 (21%)
Staph aureus	1 (7%)
Undetermined	5 (36%)
**Death rate**	8 (57.1%)
**Patients with rapid deterioration & MODS**	12 (24%)
**Delay of MODS/day**	4 (3–5)
**Death rate**	12 (100%)
**Death at day**	8 (4–11)

### 4.1 Evaluation of gene expression of BCL2 and TNFα for prediction of complications (HAI & MODS)

**Fig. 3:**
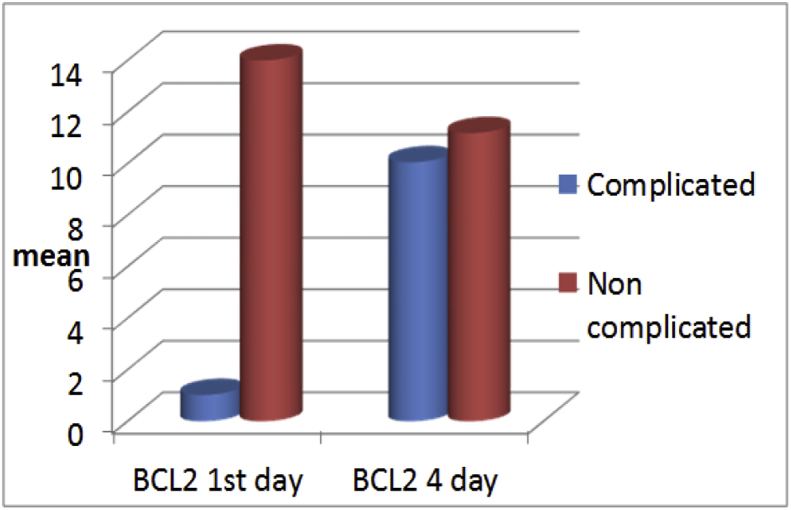
**Comparison of BCL2 gene expression levels in individual patients between day 1 and day 4 for complicated and non-complicated patients**. On day 1 sample, BCL2 is statistically lower in the HAI group than the non-HAI group (HAI/no HAI: 1.02 ± 0.7/14.50 ± 9.5, p < 0.001), While on day 4 sample, no statistical difference was observed (HAI/no HAI: 10.05 ± 6.8/11.18 ± 7.34, p < 0.06).

**Fig. 4:**
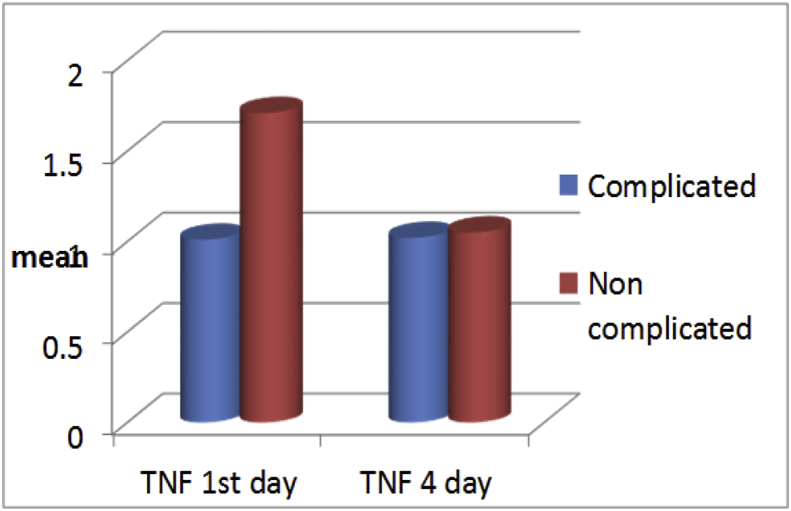
**Comparison of TNFα gene expression levels in individual patients between day 1 and day 4 for complicated and non-complicated patients**. On day1 sample, TNFα was statistically higher in non HAI group, (HAI/no HAI: 1.01 ± 0.6/1.71 ± 1.13, p < 0.02). While on day 4 sample, there was no statistical difference between groups (HAI/no HAI: 1.02 ± 0.59/1.05 ± 0.64, p = 0.64).

Comparison between gene expression in both groups in the 1^st^ and 4^th^ days of admission are presented in Fig. 3 and Fig. 4

**Fig. 5:**
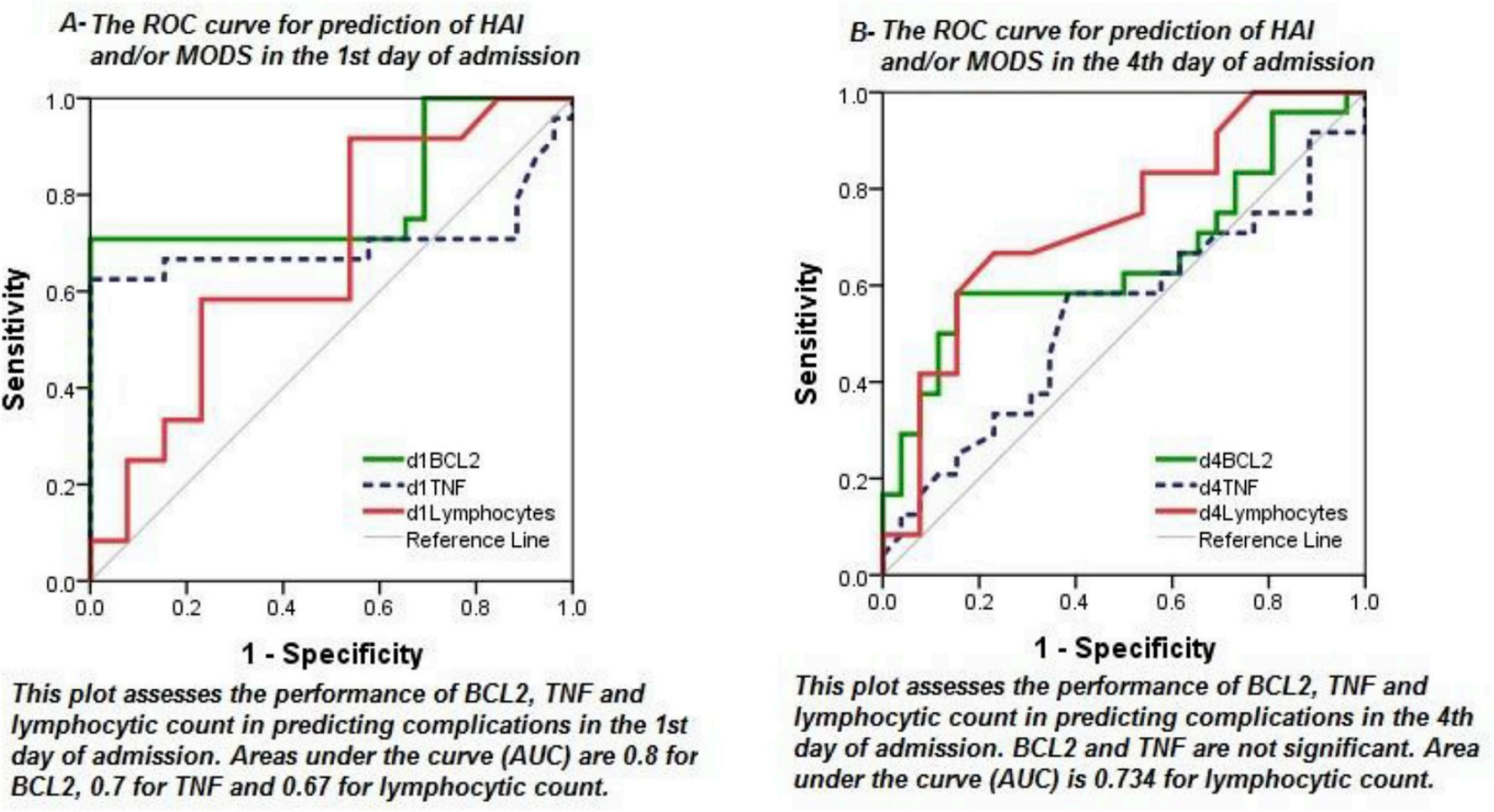
ROC curves of lymphocytes, BCL2 and TNFα in 1^st^ and 4^th^ days for prediction of complication.

**The Area under the receiver operating characteristic (ROC) curve for predicting complications**, Fig. 5: Gene expression and lymphocytic count were tested with ROC curve to detect its ability to predict complication. In the 1^st^ day: lymphocytic count, TNFα and BCL2 were significant, while in the 4^th^ day: only lymphocytic count was significant. The results of statistically significant AUC were as following:

BCL2 day 1: AUC = 0.8, At cut off value of (1.56), sens.= (76.9) specif. =(70.8).

TNFα day 1: AUC = 0.7, At cut off value of (1.53), sens.= (66.7) specif. =(84.6).

Lymph. day 1: AUC = 0.67, At cut off value of (2.55), sens.= (58.3) specif. =(76.9).

Lymph. day 4: AUC = 0.734, AT cut off value of (2.1), sens.=(66.7) specif.=(76.1).

### 4.2 Correlations between gene expression and indicators of disease severity

Pearson correlations were performed between genes expression and indicators of disease severity as OFI, PELOD, length of stay in PICU and length of stay in the hospital and described in Table 3.

**Table 3:** Pearson correlations between gene expression and PELOD, OFI, ICU and hospital length of stay.

	*1*^*st*^*day*				*4*^*th*^*day*			
	*OFI*	*PELOD*	*Length of stay in PICU*	*Length of stay in hospital*	*OFI*	*PELOD*	*Length of stay in PICU*	*Length of stay in hospital*
*BCL2*	0.565**	0.257	0.286*	0.236	0.229	0.118	0.272	0.321
*TNFα*	0.394**	0.232	0.038	0.086	0.265	0.171	0.217	0.258
*Lymph.*	0.457**	0.516**	0.211	0.234	0.393**	0.412**	0.05	0.01

*: p < 0.05, **: p < 0.01.

We observed no statistical difference in gene expression of BCL2 or TNFα in patients regarding their sex, age or body mass index.

## 5 Discussion

The immune response activation (or deactivation) can significantly affect the outcome in sepsis, trauma or any medical condition, and may vary between individuals. In patients with a similar infection, there is great variability reported in the clinical profiles and outcomes. The risk of HAI, MODS and their outcomes are influenced by host predisposition, this may be explained by genetic variability between patients [13].

Genetic studies might allow for earlier differentiation between patients with immune-inflammatory response to either infection or trauma, allowing for more focused and timely treatment. Molecular profiles can identify a good versus a poor immune response, and enable us to detect patients at risk of HAI, sepsis and MODS.

In this pilot study, we aimed to evaluate mRNA biomarkers of BCL2 and TNFα as tools for monitoring the immune response in critically ill children in our community.

Previous studies were conducted to assess the risks of HAI or MODS in critically ill patients, In our cohort, After exclusion of patients died in the first 3 days of admission, we found 2 patients categories: 1- Patients who passed the critical illness and fully recovered. 2- Patients had HAI once or more or who even progressed to MODS and died.

As immune dysregulation is the most important pathogenic mechanism underlying both HAI and MODS [13,21,22] and because of the small number of patients in this pilot study, we grouped patients who had HAI and MODS together, hoping that further studies would be with large numbers for a better analysis.

In our study, 28% of patients fulfilled the CDC criteria for the diagnosis of HAI, pneumonia was the most common cause of the 1^st^ HAI attacks, blood culture was positive in 64.3% of them and the most common organisms were Gram negative bacilli, Klebsiella and staphylococci. Twenty-four percent of patients developed septic shock, MODS and died. In previous studies, HAI rate ranged from 30% to 55% and pneumonia was the most common HAI. Blood culture positive results ranged from 35% to 55%, the most commonly isolated species were: *Klebsiella pneumoniae*, Pseudomonas, Acinetobacter, and *Staphylococcus aureus* [12,[23], [24], [25]].

We observed that surgical patients were more liable to HAI than medical and septic patients, but in previous studies, the risk was equal, with a minimal increase in complications in septic patients [12,[23], [24], [25]].

We observed that the history of previous PICU admission, delayed developmental milestones and chromosomal abnormality were associated with higher rates of complications. Also, Perronet et al., observed that children who developed HAI were more likely to have genetic or chromosomal abnormalities [12]. These risks need further assessment in a large numbered studies.

In our study, lymphocytic count was significantly lower in the patients complicated with HAI and/or MODS than the other patients in the 1^st^ day of admission and the difference increased by the 4^th^ day. In previous studies, persistent lymphopenia was highly predictive of mortality in medical [26,27], septic [28], and traumatic patients [29]. The lymphocytic count is a rapid, easy and cheap test to assess the immune state with a good predictive and follow up values.

TNFα, the proinflammatory cytokine, is tightly related to regulation of host innate immunity, inflammation, and apoptosis. In a previous study, TNFα response <200 pg/mL throughout 7 days after positive culture was associated with persistent nosocomial infection, while recovery above 200 pg/mL was associated with resolution of infection (p < 0.05) [30]. Low TNF-alpha concentration in patients with severe acute pancreatitis predicts the development of MODS and fatal outcome in another study [13]. On the other hand, there was no statistical difference between groups (HAI vs. no HAI) in their TNFα in Perronet study [12]. In another study, TNF-alpha levels were higher in non-survivors [31]. In our study, TNF-alpha gene expression was lower in the group acquired HAI or MODS but no statistical difference observed in the 4^th^ day sample. This difference can be explained as TNF-alpha is released in the circulation in the first few hours and rapidly disappears after that [13].

Lymphocytic apoptosis is recognized as the core feature of the critical illness induced immunoparalysis. Apoptosis is regulated by a wide group of pro-apoptotic and anti-apoptotic genes, BCL2 (the anti-apoptotic gene) was used to detect the enhanced lymphocyte apoptosis and identify patients at higher risk of HAI in previous studies in children and adults [[10], [11], [12]]. In our study, lower levels of BCL2 associated with HAI and worse outcome but its level returned to its normal level by the 4^t^^h^ day.

We observed that up-regulation of apoptosis and down-regulation of proinflammatory markers, which are major elements of CARS, were associated with HAI and MODS.

To our knowledge, this is the first study of mRNA biomarkers to detect the acquired immune suppression in critically ill children in our community. This study provides a new evidence that immune suppression can be measured. This finding requires confirmations in larger studies and by assessing other mRNA biomarkers which affect the immune response.

## 6 Conclusion

Nosocomial sepsis and MODS remain a major cause of morbidity and mortality in PICU. Early detection of patients at risk would be of great help to provide them with the suitable immunomodulators. Lymphocytic apoptosis is one of the causes of immunosuppression in critically ill patients. Our results showed that patients who developed HAI and/or MODS had a lower lymphocytic count, lower levels of BCL2 (the anti-apoptotic gene) and lower level of TNFα (the proinflammatory cytokine). TNFα and BCL2 on admission can predict HAI and MODS (AUC = 0.7 and AUC = 0.8), but were of no use in the follow-up, however, the lymphocytic count is a rapid, easy and cheap test to assess the immune state with a good predictive and follow up values.

## Limitations

Small sample size, heterogeneous population not classified by etiological causes.

## Recommendations

Further studies should classify patients according to their outcomes in grades and their causes of admission as surgical, medical and sepsis, with larger numbers of patients for a sufficiently powered study.

## Provenance and peer review

Not commissioned, externally peer reviewed.

## Ethical approval

Approval of the study was obtained from the Ethical Committee of Scientific Research, Faculty of Medicine, Benha University. Written informed consent was taken from the parents of each child.

## Sources of funding

This research did not receive any specific grant from funding agencies in the public, commercial, or not-for-profit sectors.

## Author contribution

Authors contributed equally in the study.

## Conflicts of interest

No conflicts of interest.

## Trial registration number

ChiCTR1800018506.

## Guarantor

The corresponding author.

## Appendix A Supplementary data

AMS-D-18-00190_DataProfile AMS-D-18-00190_DataProfile

strocss.DOC strocss.DOC
